# Single Protein Encapsulated SN38 for Tumor-Targeting Treatment

**DOI:** 10.21203/rs.3.rs-3154635/v1

**Published:** 2023-07-27

**Authors:** Changjun J. Yu, Faqing Huang, Kinsley Wang, Mengmeng Liu, Warren A. Chow, Xiang Ling, Fengzhi Li, Jason L. Causey, Xiuzhen Huang, Galen Cook-Wiens, Xiaojiang Cui

**Affiliations:** California Institute of Technology; University of Southern Mississippi Center For Tobacco Prevention and Health Promotion: University of Southern Mississippi; Sunstate Biosciences, LLC; Sunstate Biosciences, LLC; University of California Irvine Department of Medicine; Roswell Park Comprehensive Cancer Center; Roswell Park Comprehensive Cancer Center; Arkansas State University; Cedars-Sinai Medical Center; Cedars-Sinai Medical Center; Cedars-Sinai Medical Center

**Keywords:** Colorectal cancer, Single Protein Encapsulation, SN38, SPESN38, Soft tissue sarcoma, Topoisomerase I inhibitor, FcRn

## Abstract

**Background:**

The alkaloid camptothecin analog SN38 is a potent antineoplastic agent, but cannot be used directly for clinical application due to its poor water solubility. Currently, the prodrug approach on SN38 has resulted in 3 FDA-approved cancer therapeutics, irinotecan, ONIVYDE, and Trodelvy. However, only 2–8% of irinotecan can be transformed enzymatically *in vivo* into the active metabolite SN38, which severely limits the drug’s efficacy. While numerous drug delivery systems have been attempted to achieve effective SN38 delivery, none have produced drug products with antitumor efficacy better than irinotecan in clinical trials. Therefore, novel approaches are urgently needed for effectively delivering SN38 to cancer cells with better efficacy and lower toxicity.

**Methods:**

Based on the unique properties of human serum albumin (HSA), we have developed a novel single protein encapsulation (SPE) technology to formulate cancer therapeutics for improving their pharmacokinetics (PK) and antitumor efficacy and reducing their side effects. Previous application of SPE technology to doxorubicin (DOX) formulation has led to a promising drug candidate SPEDOX-6 (FDA IND #, 152154), which will undergo a human phase I clinical trial. Using the same SPE platform on SN38, we have now produced two SPESN38 complexes, SPESN38–5 and SPESN38–8. We conducted their pharmacological evaluations with respect to maximum tolerated dose, PK, and *in vivo* efficacy against colorectal cancer (CRC) and soft tissue sarcoma (STS) in mouse models.

**Results:**

The lyophilized SPESN38 complexes can dissolve in aqueous media to form clear and stable solutions. Maximum tolerated dose (MTD) of SPESN38–5 is 250 mg/kg by oral route (PO) and 55 mg/kg by intravenous route (IV) in CD-1 mice. SPESN38–8 has the MTD of 45 mg/kg by IV in the same mouse model. PK of SPESN38–5 by PO at 250 mg/kg gave mouse plasma AUC_0–∞_ of 0.0548 and 4.5007 (nmol × h/mL) for SN38 and SN38 glucuronidate (SN38G), respectively, with a surprisingly high molar ratio of SN38G:SN38 = 82:1. However, PK of SPESN38–5 by IV at 55 mg/kg yielded much higher mouse plasma AUC_0–∞_ of 18.80 and 27.78 nmol × h/mL for SN38 and SN38G, producing a much lower molar ratio of SN38G:SN38 = 1.48:1. Antitumor efficacy of SPESN38–5 and irinotecan (control) was evaluated against HCT-116 CRC xenograft tumors. The data indicates that SPESN38–5 by IV at 55 mg/kg is more effective in suppressing HCT-116 tumor growth with lower systemic toxicity compared to irinotecan at 50 mg/kg. Additionally, SPESN38–8 and DOX (control) by IV were evaluated in the SK-LMS-1 STS mouse model. The results show that SPESN38–8 at 33 mg/kg is highly effective for inhibiting SK-LMS-1 tumor growth with low toxicity, in contrast to DOX’s insensitivity to SK-LMS-1 with high toxicity.

**Conclusion:**

SPESN38 complexes provide a water soluble SN38 formulation. SPESN38–5 and SPESN38–8 demonstrate better PK values, lower toxicity, and superior antitumor efficacy in mouse models, compared with irinotecan and DOX.

## Introduction

As a topoisomerase I (Top1) inhibitor, the alkaloid SN38 (7-ethyl-10-hydroxy camptothecin) is one of the most potent cytotoxic camptothecins (CPTs) against cancer cells [1, 2]. Although SN38 has great potential to treat many malignancies, such as colorectal, lung, gastric, and ovarian cancers, it cannot be used directly in clinical applications due to its poor water solubility and spontaneous hydrolytic instability. Various prodrug approaches have been developed to solve the poor solubility problem, leading to the successful development of 3 FDA-approved drugs, irinotecan (CPT-11, 10’-OH group on ring A is conjugated to a water-soluble moiety) [3, 4], ONIVYDE (nanoliposome irinotecan) [5], and Trodelvy (antibody drug conjugate, 20’-OH group on ring E is conjugated to a sacituzumap via an acid sensitive linker, targeting the Trop-2 receptor in cancer cells) [6, 7]. The CPT derivative Irinotecan has been widely used since 1996 in advanced colorectal cancer (CRC) as a standard treatment agent in both monotherapy and combination therapy. However, only 2–8% of irinotecan [8–11] can be transformed *in vivo* into the active metabolite, SN38, and 55% of the drug is excreted as intact irinotecan in humans [12]. SN38 is 100–1000 times more cytotoxic than irinotecan [11, 13]. Therefore, irinotecan itself without SN38 transformation is inactive and has practically no therapeutic value. Irinotecan conversion into active SN38 *in vivo is* achieved by carboxylesterases in the liver [10, 14–18]. However, human liver carboxylesterase activity can vary widely among individual patients [19, 20], which can lead to patient-specific irinotecan PK [20] and antitumor efficacy. These intrinsic limitations of irinotecan significantly reduce its clinical potential [4].

To overcome these problems of using SN38 as a anticancer drug, numerous drug delivery systems, such as prodrugs, polymeric micelles, and liposome-based formulations, have been studied extensively [21]. These approaches can alter the properties of SN38, such as water solubility. The formulated SN38 has shown good efficacy against various tumors in preclinical research but showed disappointing results in the human clinical setting. SN38 liposome particles [22, 23], PEG-SN38 [24], and SN38 polymer micelle [25] did not present better antitumor efficacies relative to irinotecan in human phase II trials. Problems associated with these drug delivery systems include low drug loading, poor tumor penetration, non-targeting effects, and unfavorable drug release. Therefore, new approaches are urgently needed to formulate SN38 for higher anti-cancer efficacy and lower toxicity.

It is well-documented that human serum albumin (HSA) is a desired drug delivery carrier [26–28] due to its unique properties, such as being endocytosed and transcytosed into and across the cell via receptors [28]; long half-life of 19 days [29–32], able to accumulate at the tumor tissue due to the enhanced permeability & retention (EPR) effect; and being preferentially taken up and metabolized by cancer cells to serve as nutrients [33–37]. We previously developed the single protein encapsulation (SPE) technology to carry a predefined number of DOX (doxorubicin) molecules to form uniform HSA-DOX complexes (SPEDOXs) by an unmodified monomeric HSA molecule [32], thereby avoiding the issues associated with synthetic polymers, conjugated HSA, and HSA nanoparticles (NPs). *In vivo* studies with mice demonstrated better PK, lower toxicity, and superior tumor inhibitory activity of SPEDOXs compared with unformulated DOX [32]. The FDA has granted “Orphan Drug Designation” to SPEDOX-6 for treatment of soft tissue sarcoma (STS) patients. Phase Ib/IIa human clinical trials of SPEDOX-6 are being planned (IND# 152154).

In this study, by adopting the SPE technology, we have successfully encapsulated SN38 to create two SPESN38 complexes, SPESN38–5 (5 SN38 molecules per HSA) and SPESN38–8 (8 SN38 molecules per HSA as the maximum capacity). Preclinical evaluations using CRC and STS mouse models show that SPESN38 complexes have better PK values than those of irinotecan, resulting in 1.8-fold higher SN38 AUC_0–∞_. SPESN38 also has a higher antitumor efficacy than that of irinotecan without increased toxicity. These results demonstrate SPESN38 complexes as novel effective anticancer agents with great potential for clinical applications, thereby warranting further studies to develop them into cancer therapeutics.

## Material and Methods

### Material and instruments

HSA (25% solution) and SN38 were purchased from Octapharma USA and GLPbio Technology, respectively. Methanol, ethanol, other chemicals and suppliers were purchased from VWR. UV spectrum measurement and quantitation were conducted on a UV-1600 PC spectrometer (VWR). Both SPESN38–5 and SPESN38–5 were prepared following similar protocols for making SPEDOX-6 [32].

#### MTD studies.

All MTD studies were performed at Roswell Park Comprehensive Cancer Center Animal Facility following the animal protocol approved by the Institutional Animal Care and Use Committee (IACUC). Male and female CD-1(ICR) mice (haired) (5 to 7 weeks old) were purchased from Charles River Lab. For SPESN38–5, the lyophilized yellowish powder was dissolved in DI water to form a clear SPESN38–5 solution with light yellowish color. In PO route, the SPESN38–5 solution was fed to mice at doses of 300 mg/kg (2 female mice/group), 250 mg/kg (2 female mice/group) and 200 mg/kg (2 female mice/group) on Day 1. The percent body weight change of all mice was recorded vs days, as shown in Fig. 2A and Fig. 2B. For IV route, the SPESN38–5 solution was intravenously injected to mice at doses of 80 mg/kg (2 female mice/group), 55 mg/kg (2 female mice/group), 45 mg/kg (2 female and 2 male mice/group) and 35 mg/kg (2 female and 2 male mice/group). The percent body weight change of all mice was recorded vs days, as shown in Fig. 2C to Fig. 2F. For SPESN38–8, the same procedures were used for preparation and the resulting SPESN38–8 solution was intravenously injected to mice at doses of 45 mg/kg (2 female and 2 male mice/group) and 35 mg/kg (2 female and 2 male mice/group). The percentage of body weight change of all mice were recorded vs days, as shown in Fig. 3A and Fig. 3B.

#### PK studies.

The PK studies were performed at Roswell Park Comprehensive Cancer Center Animal Facility following the animal protocol approved by the Institutional Animal Care and Use Committee (IACUC). Female CD-1(ICR) mice (haired) (5 to 7 weeks old) were purchased from Charles River Lab. After PO administration of SPESN38–5 at the dose of 250 mg/kg to six groups (3 female CD-1 mice/group). Blood samples were collected into 1.5 mL Li-Heparin LH/1.3 tubes after anesthetizing mouse with CO_2_ are at the timepoints of 1h, 2h, 4h, 8h, 12h, and 24h (triplicate blood samples at each time point). Serum from each blood sample was obtained by 2,500 rpm centrifugation for 3 min, and the serum on the top layer was collected using pipet and transferred into 1.5 mL Eppendorf tubes and then frozen immediately in liquid nitrogen until PK analysis. After IV administration of SPESN38–5 at the dose of 55 mg/kg to six groups (3 female CD-1 mice/group) (tail vein injection), the same procedure was used for sample preparation.

For PK study, mouse plasma samples were analyzed for SN-38 and SN-38G by LC-MS/MS using a previously described method [39] over the calibration range of 0.200 to 200 ng/mL for each analyte. Briefly, an aliquot of plasma (100 uL) was mixed with acidified methanol containing the internal standards [irinotecan-d_10_ (Toronto Research Chemicals, Toronto, Canada) and camptothecin (Sigma-Aldrich, St. Louis, Missouri), respectively] for a protein precipitation extraction, followed by centrifugation and injection of the supernatant for analysis. Chromatographic separation was achieved using a Waters CORTECS C18 + LC column (100 mm × 2.1 mm, 2.7 um) maintained at 50 °C and sample elution carried out at flow rate of 300 μL/min with a biphasic gradient (water with 0.1% acetic acid and acetonitrile with 0.1% acetic acid). SN-38 and SN-38G were detected by multiple reaction monitoring (MRM) using an AB SCIEX 5500 mass spectrometer with an electrospray ionization source in positive ion mode controlled by AB SCIEX Analyst^®^ software, version 1.6.2. All sample results were obtained within one analytical run. Samples above the calibration range were diluted to be below the point of saturation of the detector.

Non-compartmental analysis (NCA) was performed utilizing mouse plasma concentrations of SN38 and SN38-G that were obtained by LC-MS/MS. Plasma samples that were included in the NCA were collected at t = 0, 1, 2, 4, 8, 12, and 24 h post-dose for PO route and at t = 0, 0.083, 1, 2, 4, 8, and 24 h post-dose for IV route. The PK parameters were calculated using Phoenix WinNonlin software: maximum plasma concentration (C_max_), Area Under the plasma Concentration-time curve (AUC), elimination half-life (t_1/2_), apparent clearance (CL/F), and clearance (CL). AUC values were calculated using the linear-up log-down method (Table 1).

### In vivo studies with the HCT-116 model

HCT-116 CRC xenograft model study was performed at Roswell Park Comprehensive Cancer Center Animal Facility following the animal protocol approved by the Institutional Animal Care and Use Committee (IACUC). HCT-1116 cell line (CCL-247) was purchased from ATCC. After growing in Eagle’s Minimum Essential, HCT-116 cells were harvested by trypsinization and washed twice with PBS. HCT-116 cells (2 × 10^6^ per injection) were suspended in 200 μl of a 1:1 solution of ice-cold PBS and Matrigel (Corning Incorporated, Corning, NY) solution. HCT-116 cancer xenograft tumors were first generated by injecting 2 × 10^6^ cancer cells into the flank area of severe combined immunodeficiency (SCID) mice (CB17SC, strain C.B-*Igh*-*1*^*b*^*/*IcrTac-*Prkdc*^*scid*^, 5 to 7 weeks old, Roswell internal breeding). After the tumors grew to 800–1200 mm^3^, they were isolated, and approximately 50 mg of non-necrotic tumor masses were subcutaneously implanted into the flank area of individual female SCID mice. When the implanted xenograft tumors grew to 250 to 350 mm^3^ on Day 7 after tumor transplantation, mice were randomly divided into 4 groups for intravenous injection: 1) vehicle (saline, 8 females), 2) SPESN38–5 (PO route at 200 mg/kg, 8 females), 3) Irinotecan (50 mg/kg, 8 females), 4) SPESN38–5 (IV route at 55 mg/kg, 8 females). The intended dose for irinotecan (pharmaceutical grade for human application) at 100 mg/kg was attempted on 2 SCID mice bearing HCT-116. Surprisingly, both died immediately. Other doses at 75 mg/kg and 50 mg/kg were tried on healthy CD-1 mice. Both mice from 75 mg/kg IV died instantly, but 2 mice from 50 mg/kg IV were safe, which is consistent with the literature report. Therefore, irinotecan treatment group only had 6 female mice for this study. Mice in group 1 and group 2 on Day 10 were sacrificed due to the large tumor size with diameter ≥ 20 mm. Tumor volume (TV) and BW were measured three times per week or daily depending on the condition of the mouse. TV was calculated using the formula: v = 0.5 (L × W^2^). Progression at the endpoint was a tumor size with diameter ≥ 20 mm or a moribund condition.

### In vivo studies with the SK-LMS-1 model

Human SK-LMS-1, leiomyosarcoma (sub-type of STS) tumor model study was performed at Roswell Park Comprehensive Cancer Center Animal Facility following the animal protocol approved by the Institutional Animal Care and Use Committee (IACUC). SK-LMS-1 cell line (HTB-88) was purchased from ATCC. After growing in Eagle’s Minimum Essential, SK-LMS-1 cells were harvested by trypsinization and washed twice with PBS. SK-LMS-1 cells (1 × 10^6^ per injection) were suspended in 200 μl of a 1:1 solution of ice-cold PBS and Matrigel (Corning Incorporated, Corning, NY) solution. SK-LMS-1 cancer xenograft tumors were first generated by injecting 1 × 10^6^ cancer cells into the flank area of SCID mice (CB17SC, strain C.B-*Igh*-*1*^*b*^/IcrTac-*Prkdc*^*scid*^, 5 to 7 weeks old, Roswell internal breeding). After the tumors grew to 800–1200 mm^3^, they were isolated, and approximately 50 mg of non-necrotic tumor masses were subcutaneously implanted into the flank area of individual mice. Equal number (12) of female and male mice were used in this experiment. When the implanted xenograft tumors grew to 250 to 350 mm^3^ on Day 7 after tumor transplantation, mice were randomly divided into 6 groups for intravenous injection: 1) vehicle (saline, 4 females), 2) DOX (5 mg/kg, 4 females), 3) SPESN38–8 (IV at 33 mg/kg, 4 females), 4) vehicle (saline, 4 males), 5) DOX (5 mg/kg, 4 males), 6) SPESN38–8 (IV at 33 mg/kg, 4 males). The intended schedule for drug or vehicle treatment was weekly for 3 doses. However, mice in groups 2 and 5 with DOX at 5 mg/kg after 2 doses lost > 20% BW, indicating severe toxicity. Mice in group 1, 2, 4 and 5 on Day 9 were sacrificed due to the large tumor size with diameter ≥ 20 mm and severe BW loss (> 20%). One male mouse from group 6 had some health issues early on and was sacrificed on Day 14. Tumor volume (TV) and BW were measured three times per week or daily depending on the condition of the mouse. TV was calculated using the formula: v = 0.5 (L × W^2^). Progression at the endpoint was a tumor size with diameter ≥ 20 mm or a moribund condition.

### Tumor tissue preparations and staining study

Tumor tissues from Sk-LMS-1 mouse study were fixed in 10% neutral buffered formalin for 24, and then transferred into 70% ethanol for up to 4 days. The fixed tissues were embedded in paraffin and sectioned at 5 microns at any time when tissues were moved into 70% ethanol. All the specimens were formalin-fixed and paraffin-embedded.

### H & E Staining

Dako CoverStainer was utilized for H & E staining analyses on the paraffin-embedded SK-LMS-1 tumor tissues with a DAKO H&E kit.

### Immunohistochemistry (IHC) analysis on Ki67 and Cleaved Caspase-3

Deparaffinized tissue sections were rehydrated and incubated in 1x pH6 citrate buffer (Invitrogen Cat #00–5000) for 20 minutes using a DAKO PT Link. With an Autostainer, the following steps and reagents were used for IHC analysis

1) Incubation in 3% H_2_O_2_ for 15 minutes; 2) Incubation with 10% normal goat serum 10 min (Thermo Fisher #50062Z) 10 min; 3) Incubation with Avidin/Biotin block (Vector Labs Cat#SP-2001); 4) Incubation with primary KI67 antibody (Abcam #ab15580 or Cleaved Caspase-3 (Asp175) antibody (Cell Signaling Cat #9661) diluted in 1% BSA for 30 min; 5) Incubation with secondary Goat anti Rabbit (Vector labs #BA-1000) for 15 min; 6) Incubation with ABC reagent (Vector Labs Cat #PK 6100) for 30 min; 7) Incubation with DAB substrate (Dako Cat #K3467) for 5 minutes; 8) Counterstained with DAKO Hematoxylin for 20 seconds; 9) Coversliped slides.

### Statistic analysis

Statistic analyses of TV and tumor weight changes are described in Supplemtary Material. Detailed statistic results are shown in **Fig. S1-S3** and **Tables S4-S10**.

## Results

**Figure 1:**
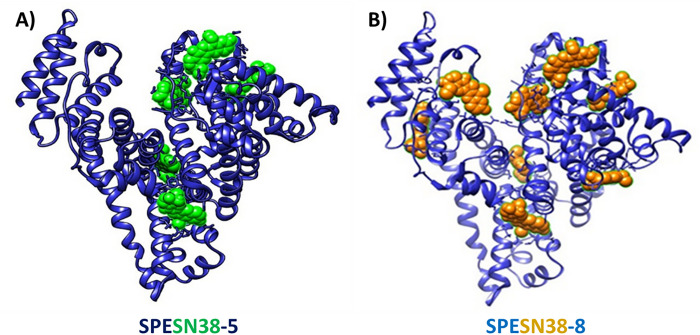
Computer docking images of SPESN38 complexes, A) SPESN38–5 and B) SPESN38–8.

Two SPESN38 complexes, SPESN38–5 and SPESN38–8 (Fig. 1), were prepared using the SPE technology [32]. The lyophilized SPESN38 complexes are highly water soluble and stable at room temperature for more than 4 h (the maximum allowable time of any injectable drug solution required by the FDA) and at 2–8°C for more than 24 h (the minimum FDA-required time of any injectable drug solution ). This report will focus on the biological evaluations of SPESN38–5 and SPESN38–8.

### MTD determination for SPESN38–5 and SPESN38–8.

**Figure 2:**
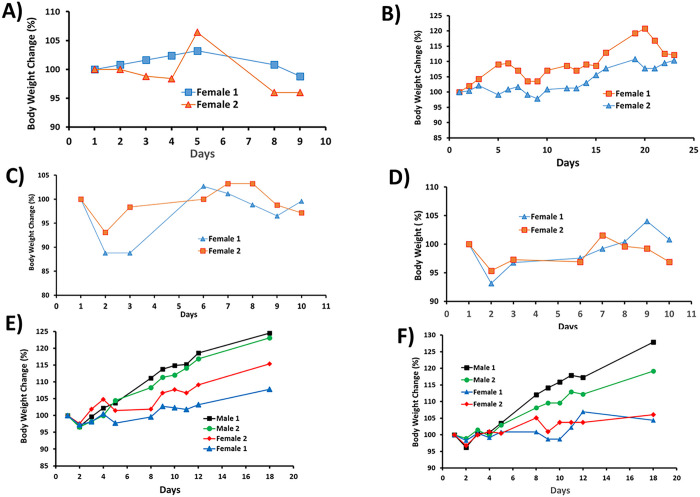
Mouse BW changes vs days after treatment for SPESN38–5 on Day 1, A) PO route at 250 mg/kg; B) PO route at 200 mg/kg; C) IV route at 55 mg/kg; D) IV route at 50 mg/kg; E) IV route at 45 mg/kg; F) IV route at 35 mg/kg.

Using CD-1 mice, we conducted MTD determination for SPESN38–5 in both oral (PO) and IV routes and SPESN38–8 in IV route. In the PO route, the first dosing at 300 mg/kg was fed to two female CD-1 mice, but one mouse died in less 72 h, indicative of exceeding MTD. The second dose at 250 mg/kg and the third dose at 200 mg/kg were evaluated and the female mouse body weight (BW) change vs the days after treatment were shown in Fig. 2A & Fig. 2B. It is clear that a 250 mg/kg dose caused a quick BW reduction after 5 days for one mouse (Fig. 2A) and a 200 mg/kg dose provided consistent results. Therefore, we concluded that the MTD for SPESN38–5 in the PO route is between 200 mg/kg and 250 mg/kg. We used a dose of 200 mg/kg for *in vivo* efficacy study and a dose of 250 mg/kg for PK study.

In the IV route for SPESN38–5, an initial dose at 80 mg/kg was attempted, both mice died immediately, meaning that the dose was too high. Lower doses at 55 mg/kg, 50 mg/kg, 45 mg/kg and 35 mg/kg were evaluated using female CD-1 mice or male/female mice. The mouse BW changes vs days after treatments were shown in Fig. 2C–2F. None of the four doses resulted in significant BW loss. Therefore, SPESN38–5 MTD at 55 mg/kg was chosen for PK and *in vivo* antitumor efficacy study.

**Figure 3:**
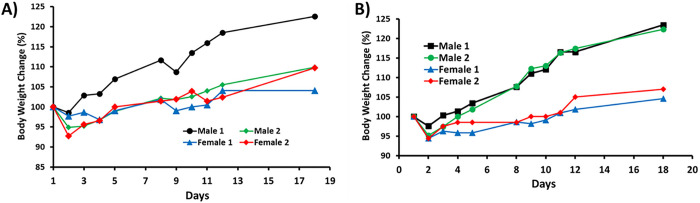
Mouse BW changes vs days after treatment for SPESN38–8 on Day 1, A) IV route at 45 mg/kg; B) IV route at 35 mg/kg.

In the IV route for SPESN38–8, two doses at 45 mg/kg and 35 mg/kg were evaluated using both male and female CD-1 mice. The mouse BW changes vs days after treatments are shown in Fig. 3A and Fig. 3B. Both doses have acceptable toxicity and 45 mg/kg for SPESN38–8 is estimated to be a single dose MTD. However, when we planned and designed *in vivo* antitumor study, we conservatively chose a dose at 33 mg/kg for 3 weekly injections in order to ensure that the body weight loss was < 20% after three doses.

### PK studies of SPESN38–5.

**Figure 4:**
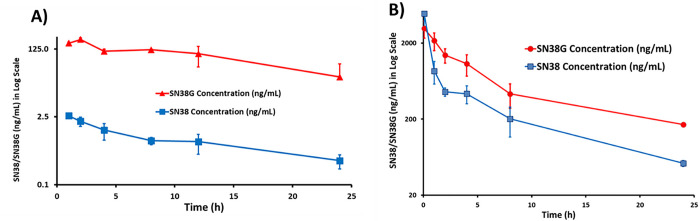
PK study of SPESN38–5 in triplicate. A) Total mouse plasma SN38 and SN38G concentrations vs times for SPESN38–5, PO route at 250 mg/kg; B) Total mouse plasma SN38 and SN38G concentrations vs times for SPESN38–5, IV route at 55 mg/kg.

A single dose of 250 mg/kg in PO route and 55 mg/kg in IV route were administered into CD-1 mice in triplicate and mouse plasma samples were collected at 1, 2, 4, 8, 12 and 24 h for the PO route and at 0.0833, 1, 2, 4, 8 and 24 h for the IV route. The total amount of SN38 and its metabolite SN38G were extracted under acidic conditions and were analyzed by LC-MS/MS. Under acidic conditions, the carboxylate forms of both SN38 and SN38G would be converted into their respective lactone forms. The total mouse serum SN38 and SN38G concentration-time profiles are shown in Fig. 4A–4B. The following PK parameters were obtained: maximum plasma concentration (C_max_), Area Under the plasma Concentration-time curve (AUC), elimination half-life (t_1/2_), apparent clearance (CL/F), and clearance (CL) (Table 1).

For the oral route at a dose of 250 mg/kg, AUC_0–∞_ for SN38 (FW = 392.40 g/mole) and SN38G (FW = 568.53 g/mole) are 21.5 and 2558.8 ng × h/mL, respectively. When converted into nmol, AUC_0–∞_ for SN38 and SN38G are 0.0548 and 4.5007 nmol × h/mL, with the molar ratio of SN38:SN38G = 1:82 in mouse plasma. It is surprising that most SN38 was glucuronidated to SN38G, an inactive form from SN38 metabolism. For the IV route at a dose of 55 mg/kg, AUC_0–∞_ for SN38 and SN38G are 7377.6 and 15794.8 ng × h/mL respectively. When converted into nmol, AUC_0–∞_ for SN38 and SN38G are 18.80 and 27.78 nmol × h/mL, with the molar ratio of SN38:SN38G = 1:1.48 (i.e. 40:60%). Therefore, the IV route at a much lower dose afforded much higher AUC_0–∞_ for SN38 and much better SN38:SN38G ratio, compared with the PO route. Based on the AUC values, the oral bioavailability of SPESN38–5 was estimated to be ~ 3%. Therefore, it is expected that antitumor efficacy of SPESN38–5 would be higher by IV over the PO route.

### Antitumor efficacy against HCT-116 tumors:

**Figure 5:**
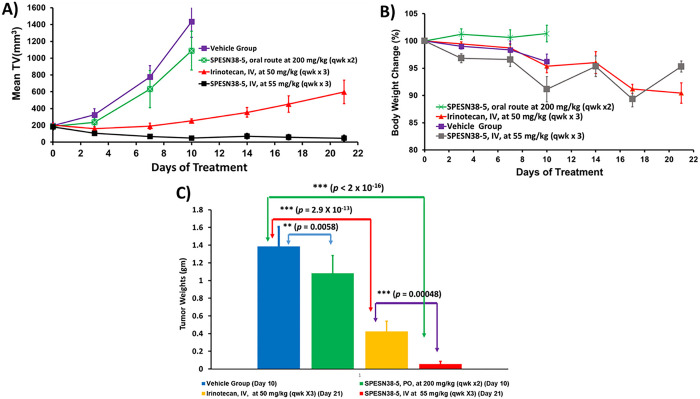
Mean TV and % BW changes vs treatment time, tumor weights for SPESN38–5 and irinotecan. A) Mean TV vs treatment time for all female mice. Mice # for each group, control (n= 8), SPESN38–5, PO (n = 8), Irinotecan (n = 6), and SPESN38–5, IV (n = 8). On Day 10, SPESN38–5, PO, TGI, 24.1%, very significantly lower than control group (***, *p* = 0.00022), Irinotecan, TGI, 82.3%, very significantly lower than control group (***, *p* < 2 ×10^−16^), SPESN38–5, IV, TGI, 96.6%, very significantly lower than control group (***, *p* < 2 ×10^−16^), SPESN38–5, IV significantly reduced tumor volume, compared to Irinotecan group (*, *p* = 0.0241). On Day 21, SPESN38–5, IV, very significantly reduced tumor volume, compared to Irinotecan group (***, *p* = 1.6 × 10^−5^); B) % BW change vs treatment time for all mice, not significantly different from each other, SPESN38–5 PO group did not display any BW loss; C) Comparison of the average tumor weights at their ending points.

Irinotecan, a prodrug of SN38, is the standard treatment of CRC. Therefore, we wanted to test the antitumor efficacy of SPESN38–5 in a CRC mouse model. The HCT-116 CRC xenograft model was chosen for the following reasons: i) its low FcRn level (< 2 TPM, transcript per million) [40]; ii) its KRAS mutation at G13D; iii) its responsiveness to irinotecan. SPESN38–5 in both PO and IV routes was evaluated, in comparison to irinotecan, against HCT-116 using female SCID mice. Due to the fast growth rate of HCT-116 tumors, all mice in the vehicle control group and SPESN38–5 at 200 mg/kg via PO had to be euthanized on Day 10 (Fig. 5A). For the irinotecan group, an intended dose at 100 mg/kg IV (a fresh GMP grade irinotecan for human injection) was attempted on 2 mice. Unfortunately, both died immediately. Other doses at 75 mg/kg and 50 mg/kg were tried on healthy CD-1 mice. Both mice from 75 mg/kg dose died instantly but the 2 mice from 50 mg/kg dose were safe, which is consistent with the literature report [41]. Therefore, MTD of irinotecan in SCID mice is 50 mg/kg via IV and was used for *in vivo* efficacy study with a total of 6 mice per group. On Day 10, tumor growth inhibition (TGI) for SPESN38–5 PO, irinotecan IV and SPESN38–5 IV, are 24.1% (***, *p* = 0.00022), 82.3% (***, *p* < 2 ×10^−16^), and 96.6% (***, *p* < 2 ×10^−16^), respectively, compared to the control group (Fig. 5A). By comparing tumor volume (TV) from Day 0 to Day 10 for each group, % TV changes are 631.8% (increase), 483.1% (increase), 20.6% (increase), −73.2% (reduction) for the control, SPESN38–5 at 200 mg/kg PO, irinotecan at 50 mg/kg IV, and SPESN38–5 at 55 mg/kg IV, respectively (**Table S1**). While SPESN38–5 via PO did slow down tumor growth (24.1% TGI on Day 10) relative to the control group, SPESN38–5 via IV shrank TV by 73.2% over the same period, indicating potent anticancer activity. In comparison, the standard CRC treatment drug irinotecan had 82.3% TGI, but the tumor still increased by 20.6% by Day 10. TV in the irinotecan group continued to increase by 135.5% from Day 10 to Day 21. In stark contrast, SPESN38–5 IV group reduced TV slightly by 3.3% (**Table S1**). The toxicity of the treatment agents was evaluated by body weight (BW) change over time, which is an established method for early-stage preclinical studies. The normalized BW changes for the above testing groups are not different from each other and all BW changes are within the acceptable ranges (< 10%) (Fig. 5B and **Table S1**). Therefore, SPESN38–5 at 55mg/kg via IV exhibited potent anticancer activity with low systematic toxicity.

The tumor tissues from all mice at their ending points were dissected and weighed (Fig. 5C). In agreement with TV measurement, the average tumor weight in the SPESN38–5 at 200 mg/kg PO group on Day 10 (**, *p* = 0.0058), irinotecan at 50 mg/kg IV group on Day 21 (***, *p* = 2.9×10^−13^) and SPESN38–5 at the 55 mg/kg IV group on Day 21 (***, *p* < 2.9×10^−16^) was significantly less than that in the control group on Day10. SPESN38–5 IV group significantly reduced tumor growth (***, *p* = 0.00048) relative to the irinotecan group on Day 21, resulting in the average tumor weight ratio of irinotecan:SPESN38–5 = 7.7:1, indicating superior SPESN38–5 efficacy over irinotecan in the CRC mouse model.

**Figure 6:**
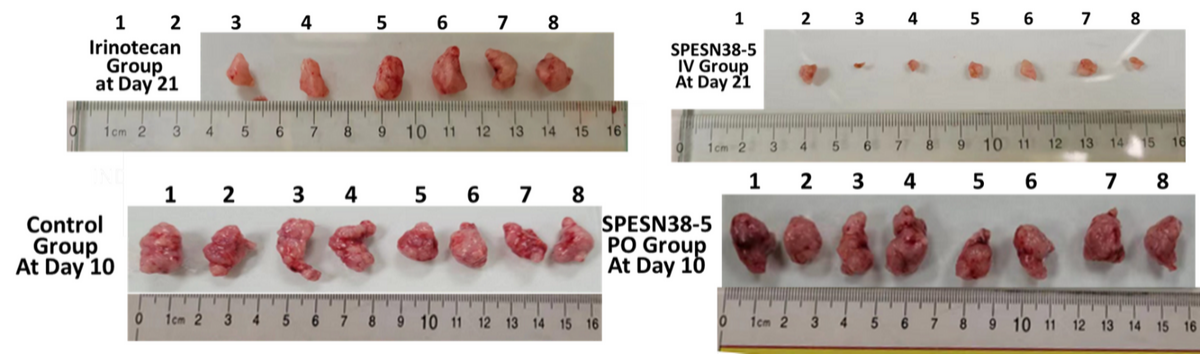
Photographic images of tumors of each group at the end of experiments. **(**Bottom), ***left,*** control groups on Day 10 when mice were euthanized due to fast tumor growth; r***ight***, SPESN38–5 PO group on Day 10. (TOP), ***left***, Irinotecan group on Day 21; ***right***, SPESN38–5 IV group on Day 21.

Photographic images of the tumors removed at the end of experiments for each treatment group are shown in Fig. 6. These images indicate that 55 mg/kg SPESN38–5 by IV route achieved much stronger antitumor effect than irinotecan at its MTD dose (50 mg/kg). Taken together, TV, tumor weight, and photographic tumor images consistently demonstrate that SPESN38–5 in IV at 55 mg/kg SN38-equivalent dose is much more effective than 50 mg/kg (MTD) irinotecan in suppressing HCT-116 tumor growth, without displaying systemic toxicity as measured by BW change. Therefore, SPESN38–5 by IV route may be a great drug candidate for further development into a clinical therapeutic against CRC and other cancers.

### Antitumor efficacy against SK-LMS-1 tumors

**Figure 7:**
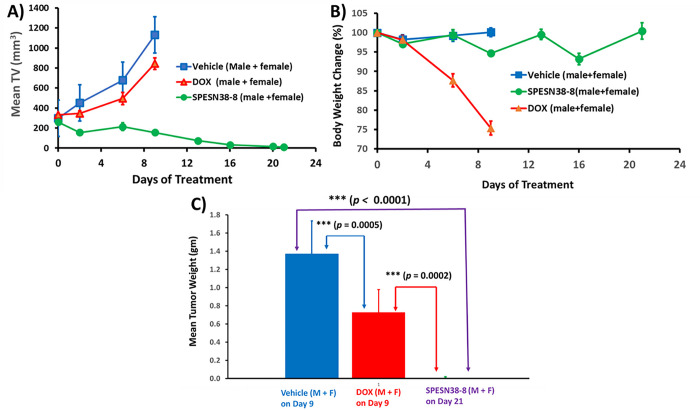
Mean TV and % BW changes vs treatment time, tumor weights for SPESN38–8 and doxorubicinfor SPESN38–8. A) Mean tumor volume vs treatment time for 4 male and 4 female mice. Mice # for each group, control (n= 8), DOX (n = 8), and SPESN38–8, IV (n = 8). On Day 6, DOX, TGI, 26.9%, significantly lower than control group (**, *p* = 0.0039), SPESN38–8, TGI, 68.5%, very significantly lower than control group (***, *p* < 0.0001). On Day 9, DOX, TGI, 25.3%, very significantly lower than control group (***, *p* < 0.0001), SPESN38–8, TGI, 86.2%, very significantly lower than control group (***, *p* < 0.0001), SPESN38–8, very significantly reduced tumor volume, compared to DOX group (***, *p* < 0.0001). On Day 21, SPESN38–8 treatment group had three mice free of tumors; B) % BW change vs treatment time for all mice. DOX group at 5 mg/kg (qwk × 2) shown severe and unacceptable toxicity, all mice have to be euthanized on Day 9. But SPESN38–8 at 33 mg/kg (qwk × 3) group did not display any BW loss; C) Comparison of the average tumor weights at their ending points.

To further explore the anticancer activities of SPESN38 complexes, we chose STS given it has limited treatment options. SK-LMS-1 as an established human leiomyosarcomas cell line is insensitive to DOX, the standard treatment for STS patients, with a high DOX IC50 of 0.49 uM [42] and has a moderate FcRn expression level (57 TPM) [40]. SPESN38–8 that encapsulated max numbers of SN38 molecules was selected for *in vivo* efficacy evaluation, in comparison to DOX against SK-LMS-1 xenograft tumors using 4 male and 4 female SCID mice per study group. This study also intended to demonstrate the superior antitumor efficacy of SPESN38–8, just like SPEDOX-5. Due to the fast growth rate of SK-LMS-1 tumors, all mice in the vehicle control group and 5 mg/kg DOX had to be euthanized on Day 9 (Fig. 7A). On Day 6, while 5 mg/kg DOX showed 26.9% TGI relative to the control group (**, *p* = 0.0039), 33 mg/kg SPESN38–8 had 68.5% TGI (***, *p* < 0.0001). On Day 9, DOX and SPESN38–8 exhibited respective 25.3% (***, *p* < 0.0001) and 86.2% (***, *p* < 0.0001) TGI, and the difference between DOX and SPESN38–8 treatment were very significant (***, *p* < 0.0001). On Day 21, 6 out of 7 mice in the SPESN38–8 treatment reached tumor-free status (One male mouse from SPESN38–8 treatment group had some health issues early on and was sacrificed on Day 14). On Day 9, TV change were 280.9% (increase) for the control group, 142.4% (increase) for the DOX treatment group, and − 40.1% (reduction) for the SPESN38–8 treatment group (**Table S2**). While 5 mg/kg DOX treatment slowed the tumor growth rate ~ 2X in TV relative to the control group, the treatment showed severe toxicity, and the mice in both the control group and the DOX treatment group had to be sacrificed on Day 9 due to the high tumor burden and unacceptable toxicity. From Day 9 to Day 21, TV in the 33 mg/kg SPESN38–8 treatment group continued to shrink to reach 95.9% (average) reduction (**Table S2**). Six of the 7 mice were tumor-free (4 mice without observable tumors and 2 mice with mouse scar tissues without tumor cells, seen the following section) at the end of the experiment (Day 21).

The normalized BW change for 5 mg/kg DOX treatment group is < 80% (75.4%), indicating unacceptable toxicity without effective antitumor activity (Fig. 7B and **Table S2**). In contrast, 33 mg/kg SPESN38–8 treatment did not show any systemic toxicity (100.4% normalized BW changes on Day 21) with excellent anticancer activity (Fig. 7A and **Table S2**). The combined data from BW change and TV confirm that DOX is not suitable for treating SK-LMS-1 (leiomyosarcoma). SPESN38–8 with a different mechanism of action (Topo I inhibitor) can overcome the resistance of some subtypes of STS toward DOX (Topo II inhibitor) treatment.

The tumor tissues from all mice at their ending point were dissected and weighed (Fig. 7C). In agreement with TV, the average tumor weight in DOX group (***, *p* = 0.0005) on Day 9 and in SPESN38–8 group (***, *p <* 0.0001) on Day 21 was significantly less than that in the control group on Day 9. In addition, the SPESN38–8 group on Day 9 significantly reduced the tumor growth (***, *p* = 0.0002), relative to the DOX group on Day 9. At the end of the experiment (Day 21), SPESN38–8 treatment led 4 mice free of tumor, with other 2 mice having tumor appearance but later proved to be mouse scar tissues without tumor cells by immunohistochemical (IHC) staining (see below section).

**Figure 8:**
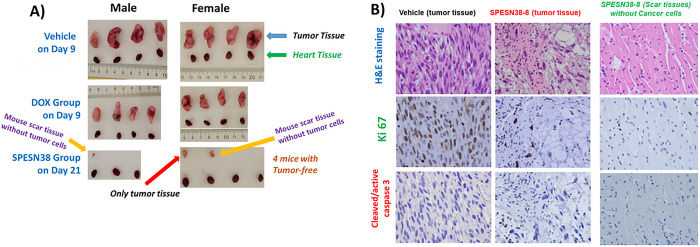
Photographic images of tumors and hearts and Immunohistochemical staining (ICS) images for SPESN38–8. A) Photographic images of tumors and hearts of each group at the end of experiments. ***Top***, control groups on Day 9 when mice were euthanized due to fast tumor growth; ***Middle***, DOX group on Day 9 when mice were euthanized due to severe toxicity, ***Bottom***, SPEDSN38–8 group on Day 21, having six mice with no tumors or scar tissues without tumor cells, B) Immunohistochemical staining images (40X) of paraffin-embedded tumor tissues (SK-LMS-1)sections for H & E, Ki67 and cleaved/active caspase 3 for tumor tissues for control group *(left panel)* and tumor tissue from SPESN38–8 treatment group *(middle panel)* and scar tissue without cancer cell from SPESN38–8 treatment group *(right panel).*

Photographic images of tumors and heart removed at the end of experiments for each treatment group are shown in Fig. 8A. These images clearly indicate that 33 mg/kg SPESN38–8 achieved great antitumor effect relative to DOX at its MTD dose (5 mg/kg). To further assess treatment effect on tumors, the dissected SK-LMS-1 tumor tissues from the control group (8 tissues) and the SPESN38–8 treatment group (3 tissues) were fixed and paraffin embedded for IHC studies. The dissected tumor tissues from the DOX treatment group were not fixed for further study because they were similar to the tumor tissues from the control group due to the insensitivity of DOX treatment to SK-LMS-1. The paraffin-embedded tumor tissue sections were subjected to H&E staining (tissue morphology), Ki67 staining (a cellular marker for proliferation), and cleaved/active caspase 3 staining (a marker for programmed cell death). H&E, Ki67, and cleaved/active caspase 3 staining on one tissue from the control group and the SPESN38–8 treatment groups with tumor tissue or mouse scar tissue were shown in Fig. 8B. On the left panel, the control tumor tissue displayed high cancer cell density with large nuclei and high Ki67 but low cleaved caspase 3 levels. In the middle panel, a representative tumor tissue of the SPESN38–8 treatment group from the bottom panel of Fig. 8A from showed reduced cancer cell density and lower Ki67 but higher cleaved caspase 3 levels relative to the left panel, indicating the antitumor effect by SPESN38–8. On the right panel, surprisingly, cancer cells were not detected in scar tissue samples from the bottom panel of Fig. 8A. Of note, the xenograft models for efficacy studies were not derived directly from human cancer cell injections but from implantation of tumor tissue fragments from earlier injections of cancer cells. In this procedure, mouse scar tissues may sometimes appear at the tumor implantation site that look like a small tumor.

Taken together, TV, tumor weight, photographic tumor images, and IHC staining results convincingly demonstrate that SPESN38–8 at 33 mg/kg (SN38-equivalent) dose is very effective in suppressing SK-LMS-1 STS tumor growth with low systemic toxicity, eliminating the implanted tumor from 6 out of 7 mice while significantly reducing the tumor size of the other (Fig. 8). Therefore, SPESN38–8 as a novel form of unmodified SN38 displays highly desirable drug-like properties, such as increased MTD, PK values, and antitumor efficacy. It is a promising drug candidate that warrants further preclinical and clinical studies for developing it into an efficacious drug against CRC, STS, and other cancers.

## Discussion

CPTs belong to class of TOP1 inhibitors [2, 45–51]. Among CPTs [44], SN38 stands out as one of the most potent cancer therapeutics. It is well known that the lactone form (L form) of CPTs undergoes pH-dependent and reversible ring opening through hydrolysis [52] to produce the inactive carboxylate form (C form) [53]. Different CPTs have similar t_1/2_ for the ring opening reaction and reach the equilibrium state with ~ 20/80% L/C forms in PBS buffer at 37°C. However, the presence of HSA significantly changes the kinetics and thermodynamics of the ring opening reaction, due to differential HSA binding to L/C forms of different CPTs. While HSA binds the L/C forms of CPT with a 157-fold higher affinity for the C form, SN38 binding to HSA is reversed, with 4.3-fold stronger binding for the L form. As a result, HSA increases the ring opening of CPT with decreasing t_1/2_ and < 0.5% L form at the equilibrium. On the contrary, SN38’s t_1/2_ and the L form at the equilibrium both increase substantially in the presence of HSA, from 20 min to 35 min and 13–38%, respectively [53]. For the past decades, the unique properties of SN38 attracted many research attempts, but its poor aqueous solubility has hindered its development as an unmodified drug.

Consequently, the prodrug approach led to an irinotecan approved by the FDA, generating active metabolite SN38 by a biotransformation [10, 14–18]. The complex PK and low fraction conversion (2–8%) of irinotecan in human setting [8–11] have resulted in inconsistent PK behaviors and efficacy among different patients [19, 20].

The current study represents the first example of developing unmodified SN38 in soluble and stable forms for *in vivo* antitumor evaluation. Based on the similar preparation procedure except for different SN38/HSA ratios, SPESN38–5 and SPESN38–8 are expected to display similar pharmacological properties such as MTD, PK, and antitumor efficacy. Toxicity study in mice indicated SPESN38–5 and SPESN38–8 with respective 55 and 45 mg/kg MTD, confirming their similar but not identical properties. We thus proceeded to conduct PK and different tumor model studies with either SPESN38–5 or SPESN38–8. SN38 AUC_0–∞_ of SPESN38–5 at a single IV dose of 55 mg/kg (Table 1) is similar to that of irinotecan at a dose of 200 mg/kg (**Table S3**) [59]. Based on their molecular weights, the SN38 AUC_0–∞_ value for SPESN38–5 is estimated to be 1.8 times higher than that for equivalent irinotecan. Since the carboxylesterase activity is much lower in humans than in mice [54], much smaller % irinotecan biotransformation to SN38 in humans relative to mice is expected, likely resulting in much lower SN38 AUC_0–∞_ value of irinotecan compared to SPESN38–5 in human plasma. Unlike the prodrug irinotecan, SPESN38–5 does not need biotransformation to SN38 by carboxylesterases, minimizing insistency among different patients. As demonstrated for SPEDOX-6 [32], delivery of SPESN38–5 to cancer cells via endocytosis, followed by SN38 dissociation and/or HSA hydrolysis by proteases, releases unmodified SN38 into the cytosol of cancer cells. Furthermore, while HSA has a long half-life of 3 weeks in human serum, due to its effective rescue and recycling through strong HSA-hFcRn (human FcRn) binding, MSA-mFcRn (mouse serum albumin-mouse FcRn) binding is weak [60], leading to a short HSA half-life in mice. As a result, SPESN38–5 is expected to have even better SN38 AUC_0–∞_ value relative to irinotecan in humans than in mice.

As expected from the PK values, both SPESN38–5 and SPESN38–8 demonstrated excellent antitumor efficacy in 2 mouse models. In the HCT-116 CRC model, SPESN38–5 at 55 mg/kg showed superior antitumor activity compared to irinotecan (**Table S1**). Separately, in the SK-LMS-1 STS model, excellent antitumor activity was achieved by SPESN38–8 at 33 mg/kg, resulting in 6 of 7 mice tumor-free. In stark contrast, conventional DOX at 5 mg/kg (MTD) was ineffective. Due to a fact that each HSA molecule in SPESN38–8 carries 60% more of SN38 than SPESN38–5 without lowering anticancer efficacy and higher toxicity, the proposed plan of SPESN38–8 (IND #: 164346) in the IV route via a 505 b (2) regulatory pathway was green-lighted by the FDA and the IND-enabling studies are under way. Furthermore, SPESN38–5 in the PO route did not provide a viable option for treating cancer because of low oral bioavailability.

It is known that tumor cells aggressively take up HSA as nutrients to support fast growing tumor cells [33–35, 37]. As such, SPESN38–5 and SPESN38–8 may achieve targeted SN38 delivery to cancer cells due to: 1) HSA (in SPESN38) is taken up by tumor cells, and dissociation and/or enzymatic degradation of HSA release SN38; 2) the acidic tumor microenvironment destabilizes SPESN38 as demonstrated for SPEDOX-6 [32], resulting in HSA’s conformation change and liberation of SN38; 3) Secreted Protein Acidic and Rich in Cysteine (SPARC) with binding affinity to HSA, may play an important role in promoting tumor uptake of HSA and ABRAXANE [62, 63], although recent clinical trials did not reveal a significant correlation between SPARC expression and the treatment outcome of ABRAXANE [64].

The long half-life of HSA can be attributed to FcRn-mediated rescue and recycling mechanism [29–31, 65]. If cancers express less FcRn, they are expected to have less HSA (SPESN38) recycling capacity, leading to increased endocytosis, SN38 dissociation, and lysosomal degradation of HSA (SPESN38). Consequently, cancer cells would get higher concentrations of SN38 relative to normal cells in cancer patients. Published reports [33, 35–37, 66] and a database [67] convincingly show that many types of cancer, including breast cancer, lung cancer, cervical cancer, ovarian cancer, pancreatic cancer [38], CRC [37], have significantly lower levels of FcRn, which promotes tumor growth by increasing HSA endocytosis and consumption. Therefore, FcRn expression levels might offer a promising cancer-targeting strategy for development of HSA-encapsulated drugs for attacking various cancers [68].

Conventional drug-containing NPs are usually assembled from lipids, synthetic and natural polymers, and inorganic materials. These NPs can be made in different size ranges and are heterogeneous in size distribution (polydisperse). Furthermore, drug molecules are often linked to the carrier through covalent conjugation. In contrast, the SPE technology has the following unique properties: 1) The formulation process involves no chemical steps. HSA encapsulation of drug molecules are achieved through multiple non-covalent interactions between HSA and drug molecules under a specific set of conditions; 2) The binary system contains a single native HSA molecule that encapsulates a predefined number of a specific drug molecule in its unmodified form; 3) The resulting SPEDRUG complex is uniform in size (monodisperse) and has the same size of a native HSA molecule; 4) The number of drug molecules per HSA molecule may be adjusted according to specific application; 5) The HSA-drug binding strength is tunable by adjusting formulation conditions to effect PK and antitumor efficacy. The successful development of SPEDOX-6 [32], SPESN38–5, and SPESN38–8 has demonstrated the utility and versatility of the SPE platform. We are actively developing other SPE-Drug complexes, and different SPEDRUG complexes are expected in the future.

## Conclusion

Using the newly developed SPE technology, we prepared SPESN38–5 and SPESN38–8, demonstrating the first examples of unmodified SN38 in clear, stable, and injectable solution. Compared with irinotecan and UF DOX in animal models, SPESN38–5 and SPESN38–8 showed favorable pharmacokinetic values, superior antitumor efficacy against CRC and STS, and lower systemic toxicity. The successful development of SPEDOX-6, SPESN38–5, and SPESN38–8 has validated the SPE platform in drug formulation. These SPEDRUG complexes represent a new uniform macromolecular nanodrug that may be used to target low FcRn expressing cancer cells, further improving their antitumor efficacy while reducing side effect toxicities. These promising preclinical results have prompted these SPEDRUG complexes to be aggressively pursued for their clinical applications.

## Figures and Tables

**Table 1:** Summary of PK parameters for SPESN38-5

	C_max_ (ng/mL)	AUC_0 – 24h_ (ng × h/mL)	AUC_0–∞_ (ng × h/mL)	T_1/2_ (h)	CL/F L/h/kg)	CL (mL/h/kg)
**Single PO dose of SPESN38-5 (250 mg/kg)**						
SN38	2.65	17.4	21.5	11.3	11619	-
SN38-G	216.33	2312.1	2558.8	6.9	-	-
**Single IV dose of SPESN38-5 (55 mg/kg)**						
SN38	4883.33	6852.3	7377.6	6.9	-	7455
SN38-G	3123.33	13931.8	15794.8	7.6	-	-

## Data Availability

Data is available on reasonable request. All data generated or analyzed during this study are included either in this article or in the supplementary information files.
